# Mammalian sterile 20 kinase 1 and 2 are important regulators of hematopoietic stem cells in stress condition

**DOI:** 10.1038/s41598-018-19637-y

**Published:** 2018-01-17

**Authors:** Da-Hye Lee, Tae-Shin Kim, Dongjun Lee, Dae-Sik Lim

**Affiliations:** 1 Department of Biological Sciences, National Creative Research Initiatives Center, Korea Advanced Institute of Science and Technology, Daejeon, Korea; 2 Department of Medical Science, Pusan National University School of Medicine, Yangsan, 50612 Korea

## Abstract

The mammalian Hippo signaling pathway has been implicated in the self-renewal and differentiation of stem and progenitor cells. MST1 and MST2 (MST1/2) are core serine-threonine kinases in the Hippo signaling pathway, one of which, MST1, has been extensively investigated for its role in T cell and myeloid cell function. These studies have identified MST1 as a promising therapeutic target in immunological disease. However, the roles of MST1/2 in hematopoietic stem cell (HSC) function *in vivo* are not fully understood. Here, we report that mice with a conditional deletion of *Mst1/*2 exhibit impaired hematopoietic stem and progenitor cell (HSPC) function under stress condition. Furthermore, *Mst1/2* deletion markedly altered mature cell output. Therefore, MST1/2 are indispensable for maintenance as well as function of stem and progenitor cells under steady state conditions and with transplantation stress.

## Introduction

Hematopoietic stem cells (HSCs) mainly reside in the microenvironments in the bone marrow (BM), where they pass through multiple developmental steps to produce mature blood cells^1,2^. During hematopoietic stresses, such as injury, infection and niche disruption, HSCs become activated and participate in tissue generation, maintenance, and repair^2,3^. Under homeostatic conditions, HSCs preserve the potential for long-term self-renewal that retains stemness during division^4^ and the capacity for subsequent reconstitution^5^. However, under stress condition such as serial transplantation, HSCs can lose their capacity for self-renewal and reconstitution, a phenomenon referred to as stem cell exhaustion^5^. For the successful bone marrow transplantation, HSCs have to be engrafted into bone microenvironment and then be expanded. In other words, the key factors for reconstitution of HSC transplantation is the efficiency of HSC engraftment/homing and retention in the BM niche^6^. HSC homing and engraftment depend on the chemotactic axis^7^.

The Hippo pathway regulates the self-renewal and differentiation of stem and progenitor cells, and plays key roles in controlling organ size and regeneration^8–12^. Mammalian sterile-20 kinase 1 and 2 (Mst1/2), mammalian homologs of Hippo, are a core pair of serine-threonine kinase in the Hippo signaling pathway that regulate the cell cycle and apoptosis^13–16^. MST1/2 have also been implicated in hepatocellular sarcoma, intestinal adenocarcinoma, and lymphoma. *Mst1/2* are expressed in most organs as well as the hematopoietic system^17^. Early studies identified an important role for MST1 and RAPL, an alternatively spliced form of RASSF5 (Ras association domain family member 5) that interacts with MST1, in lymphocyte trafficking and migration through integrin signaling^18^. Studies of *Mst1*-deficient mice have demonstrated that MST1 regulates the production of reactive oxygen species (ROS) through FOXO3A (forkhead box O3A), thereby preventing apoptosis of peripheral T cells^14^. In addition, it was recently shown that MST1/2 are capable of regulating NF-κB signaling in myeloid cells under conditions of infection^19^. MST1/2 also participate in the regulation of hematopoietic and endothelial progenitors in *Xenopus*^20^. These observations indicate that MST1/2 are important in hematopoietic cells; however, little is known about the roles of MST1/2 in HSC regulation.

In this study, we found that Mst1/2 control the production of mature hematopoietic cells. In addition, HSCs were functionally impaired in mice in which *Mst1/2* were conditionally deleted, as evidenced by alterations in the steady-stated HSC population in BM and impaired function of *Mst1/2*-deficient HSCs under stress conditions. *Mst1/2*-deficient cells also showed a poor ability to engraft into BM compared with control cells. These data suggest that inefficient engraftment of *Mst1/2*-deficient BM cells in a transplantation setting is attributable to decreased homing ability. Collectively, our findings suggest that MST1 and MST2 regulate HSC function.

## Material and Methods

### Animal studies

*Mst1*^*Floxed/Floxed*^ mice, kindly provided by Dr. T. Kinashi (Kansai Medical University), were interbred with conventional *Mst2*-knockout mice, after which *Mst1*^*fl/fl*^*;Mst2*^*-/-*^*;Mx1-Cre* mice were generated by crossing with a *Mx1-Cre*strain mouse^21,22^. For induction of Cre expression, *Mst1*^*fl/fl*^*;Mst2*^*-/-*^*;Mx1-Cre* mice were intraperitoneally (i.p.) injected with pIpC (polyinosinic–polycytidylic acid) every 2 days for 2 weeks^23,24^. For BM transplantation, lethally irradiated recipient mice were intravenously (i.v.) transplanted with competitor BM cells (0.5–2×10^6^) from CD45.1 mice and test BM cells (0.5–2×10^6^) from control or *Mst1/2* double-knockout (DKO) mice. All mice were kept in a specific pathogen-free facility at Korea Advanced Institute of Science and Technology (KAIST). The Institutional Animal Care and Use Committee of KAIST approved all of the following research protocols (approval ID: KA2010–23), including the surgical procedures and animal care, and all methods were performed in accordance with the relevant guidelines and regulations.

### Flow cytometric analysis

Flow cytometry was performed as described previously^24^. BM cells were collected from femurs and tibias by flushing with fluorescence-activated cell sorting (FACS) buffer, consisting of phosphate-buffered saline (PBS) containing 2% fetal bovine serum (FBS) and 0.1% sodium azide. Splenocytes were obtained by mincing spleens on a 40-μm cell strainer with FACS buffer. Peripheral blood cells were collected from the tail vein or heart. White blood cell preparations were obtained after lysing red blood cells with an ammonium-chloride-potassium (ACK) lysis buffer. White blood cells were detected with biotin-conjugated antibodies against CD4 (RM4–5), CD8a (53–6.7), GR1 (RB6–8C5), CD11b (3A33), B220 (RA3–6B2), Nk1.1 (PK136) and TER119; LSKs (Lin^−^Sca1^+^cKit^+^ cells) were detected using Sca1-PE-Cy7 (D7) and cKit-APC (2B8) antibodies.

### BrdU incorporation assay

Cells were obtained from mice 4 hours after injecting with BrdU (100 mg/kg, i.p.). Cells were then fixed, permeabilized and immunostained for cell surface markers (as detailed above) and BrdU using a BrdU FITC kit (BD Biosciences) as per the manufacturer’s instructions.

### Short-term engraftment assay

Short-term engraftment assays were performed by transplanting 3 × 10^7^ BM cells from *Mst1/2*-deficient or control (CD45.2^+^) mice into CD45.1^+^ mice. Three weeks after transplantation, donor cell chimerism was examined in BM, spleen, and thymus.

### Homing assay

Homing assays were performed by transplanting carboxyfluorescein succinimidyl ester (CFSE)-labeled BM cells (4 × 10^6^) from *Mst1/2*-deficient or control mice into lethally irradiated recipient mice. Sixteen hours after transplantation, CFSE^+^ cells were assessed in BM, spleen, and peripheral blood (PB).

### Colony Formation Assays

For assessing hematopoietic progenitor cell activity, BM, spleen, and PB cells were counted and plated in methylcellulose medium (M3434, STEMCELL Technologies)^24^. The colony number is counted 7 days after plating.

### Statistics

Sample sizes required for experiments were estimated based on preliminary results. No blinding or randomization was performed for any of the experiments. The statistical significance of differences between population means was assessed by two-tailed unpaired Student’s t-test, unless otherwise indicated. Statistically significant differences (*p ≤ 0.05, **p ≤ 0.01) for pairwise comparisons between the indicated data points are shown. Statistical comparisons between individual data points and groups were not performed. The Kaplan-Meier log-rank test was used to analyze mouse survival data.

## Results

### Effects of *Mst1/2* conditional deletion on mature cell subsets under homeostatic conditions

**Figure 1:**
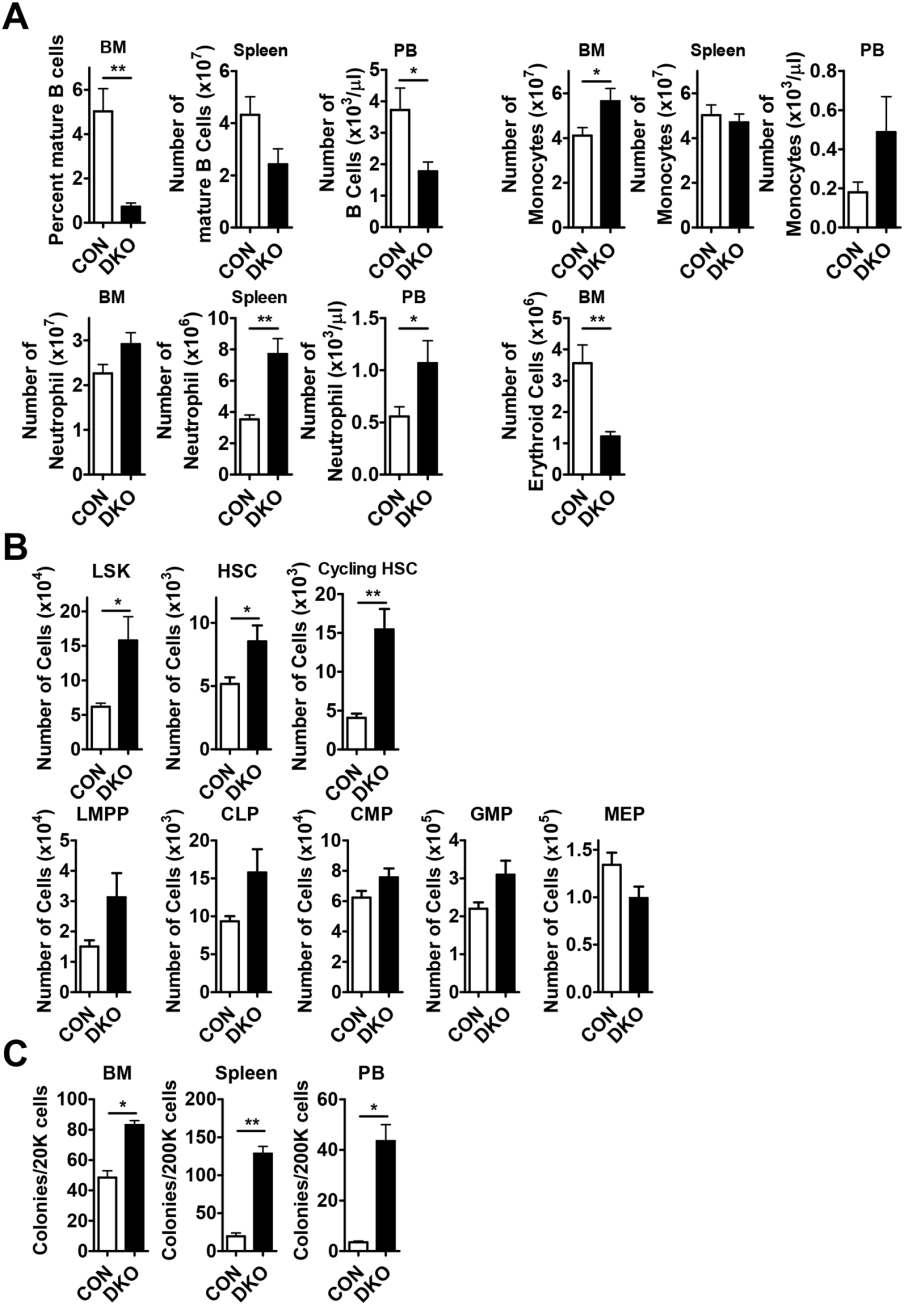
Pools of HSCs and progenitors are increased in *Mst1/2*-DKO mice. **(A)** The frequencies of B cells (B220) and myeloid cells (monocytes, Mac1^+^Gr1^lo^; neutrophils, Mac1^+^Gr1^+^) in the BM, spleen and PB, and erythroid cells (TER119) in the BM, from mice of the indicated *Mst1/2* genotypes are shown. **(B)** The frequencies of LSK cells, HSCs and cycling HSCs were decreased in BM from *Mst1/2*-DKO mice. The frequencies of committed progenitors (LMPPs, CLPs, CMPs, GMPs and MEPs) were increased in BM from *Mst1/2*-DKO mice. **(C)** The total numbers of colonies produced in M3434 media from cells of mice of each genotype are indicated. Error bars indicate S.E.M. (*p ≤ 0.05, **p ≤ 0.01, n = 4).

To investigate the role of MST1 and MST2 in HSPCs, we crossed *Mst1*^*floxed/floxed*^;*Mst2*^−/−^ mice with *Mx1*-*Cre* transgenic mice^21,22^ in which Cre recombinase is activated by administration of pIpC^24^. Consistent with previous studies on lymphopenia^14^, B cells were dramatically decreased in the BM, spleen and peripheral blood (PB) from *Mst1*^floxed/floxed^;*Mst2*^−/−^;*Mx1*-*Cre* mice (referred to as DKO; Fig. 1A). However, mild T cell lymphopenia was detected (data not shown). We also found that DKO mice showed erythropenia in the BM and myeloid expansion in BM, spleen, and PB (Fig. 1A).

The absolute numbers of Lin^−^Sca1^+^cKit^+^ cells (LSKs), HSCs (Lin^−^Sca1^+^cKit^+^CD150^+^CD48^−^), and cycling HSCs (Lin^−^Sca1^+^cKit^+^CD150^+^CD48^+^) among BM cells from DKO mice were greater than those from control mice (Fig. 1B and Fig. S2). In addition, lymphoid-primed multipotent progenitors (LMPPs; Lin^−^ Sca1^+^cKit^+^CD34^+^FLT3^+^), common lymphoid progenitors (CLPs; Lin^−^ Sca1^low^cKit^low^ CD127^+^), common myeloid progenitors (CMPs; Lin^−^Sca1^−^cKit^+^CD34^+^CD16/32^−^) and granulocyte-macrophage progenitors (GMPs; Lin^−^Sca1^−^cKit^+^CD34^+^CD16/32^+^) among BM cells from DKO mice were also increased in number, exhibiting increases comparable to or slightly less than those of HSCs (Fig. 1B). *Mst1/2* deletion also led to a decrease in megakaryocyte-erythroid progenitors (MEPs; Lin^−^Sca1^−^cKit^+^CD34^−^CD16/32^−^) in the BM and a concomitant decrease in the number of erythroid cells in the BM (Fig. 1A,B). Finally, *Mst1/2*-deficient progenitors could grow *ex vivo* into colonies than that of control (Fig. 1C).

This HSC expansion phenotype also manifested in conventional *Mst1-*knockout mice. Notably, the absolute numbers of each HSC compartment in *Mst1*-deficient mice were also increased owing to a similar or slightly increased number of total BM cells compared with that in wild-type (WT) mice (Fig. S1A). However, conventional *Mst2*-knockout mice showed a modest increase in HSCs and progenitors (Fig. S1B). These results suggest that MST1/2 could function to limit the size of the HSC pool.

### The expansion of HSC-enriched populations in *Mst1/2*-DKO mice is caused by increased proliferation and decreased apoptosis

**Figure 2:**
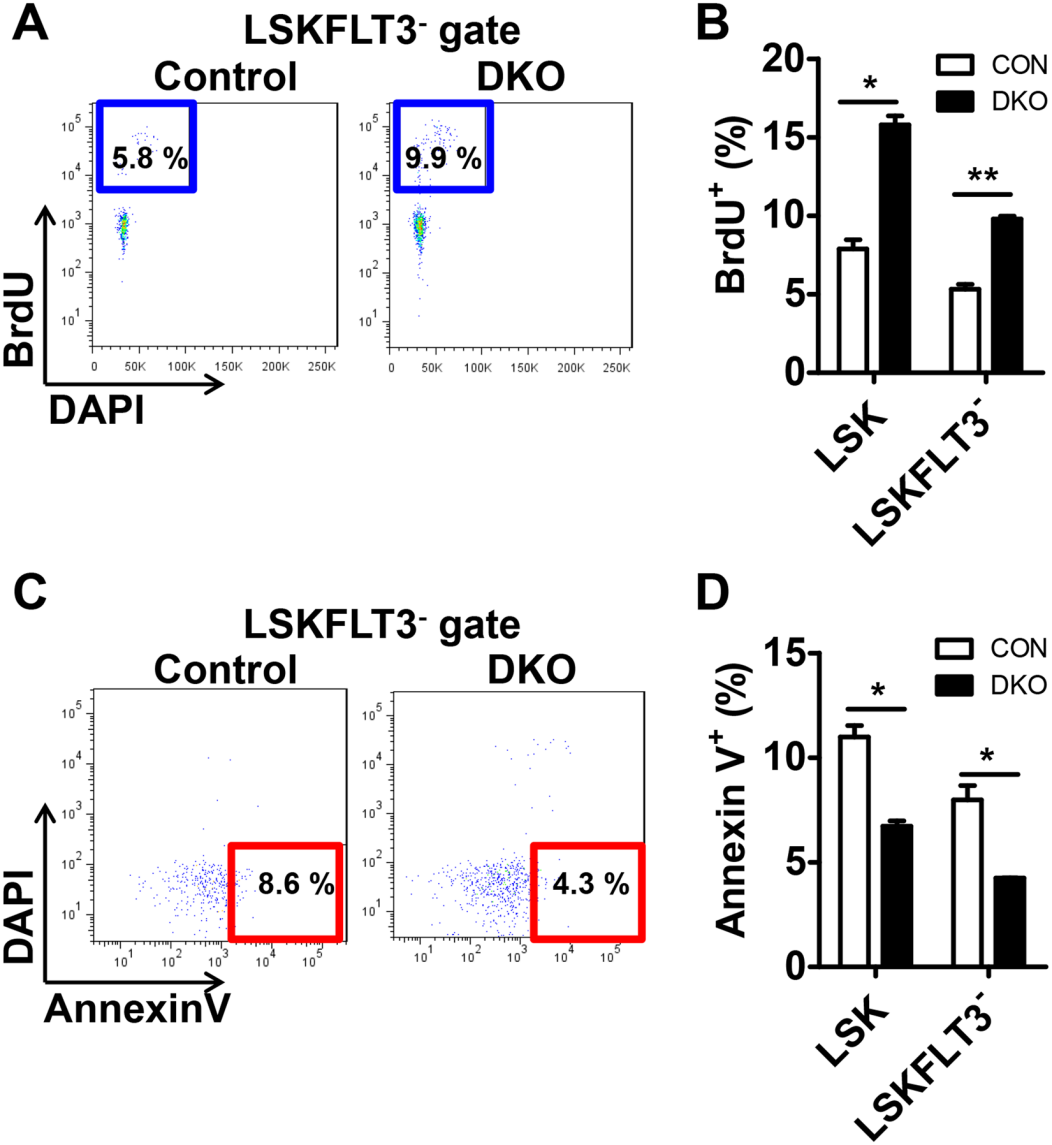
The frequency of proliferation is increased and the frequency of apoptosis is decreased in *Mst1/2*-DKO HSCs. **(A)** FACS plot showing BrdU-incorporating populations in [LSK]FLT3^−^ cells. **(B)** *Mst1/2*-deficient HSCs showed greater proliferation than controls. **(C)** FACS plot showing annexin V^+^/DAPI^−^ populations in [LSK]FLT3^−^ cells. **(D)** *Mst1/2*-deficient HSCs showed less apoptosis than control cells. Error bars indicate S.E.M. (*p ≤ 0.05, **p ≤ 0.01, n = 2).

To determine the cause of increased HSCs, we performed BrdU-incorporation assays *in vivo*. The proliferative activity of the HSC compartments, LSK and LSKFLT3^-^, were both increased in *Mst1/2*-DKO mice (Fig. 2A,B). It has been reported that MST1/2 kinases are crucial in mediating/promoting apoptosis under conditions that stimulate apoptosis^17^. Therefore, we analyzed whether altered apoptosis could contribute to the expansion of HSCs in *Mst1/2*-DKO mice. We found that there were fewer apoptotic (annexin V^+^/DAPI^−^) cells in HSC compartments from the *Mst1/2*-DKO mouse (Fig. 2C,D). These data indicate that MST1/2 maintain HSC pools *in vivo* through regulation of proliferation and apoptosis.

### HSCs were not exhausted in 6-month-old *Mst1/2*-DKO mice

**Figure 3:**
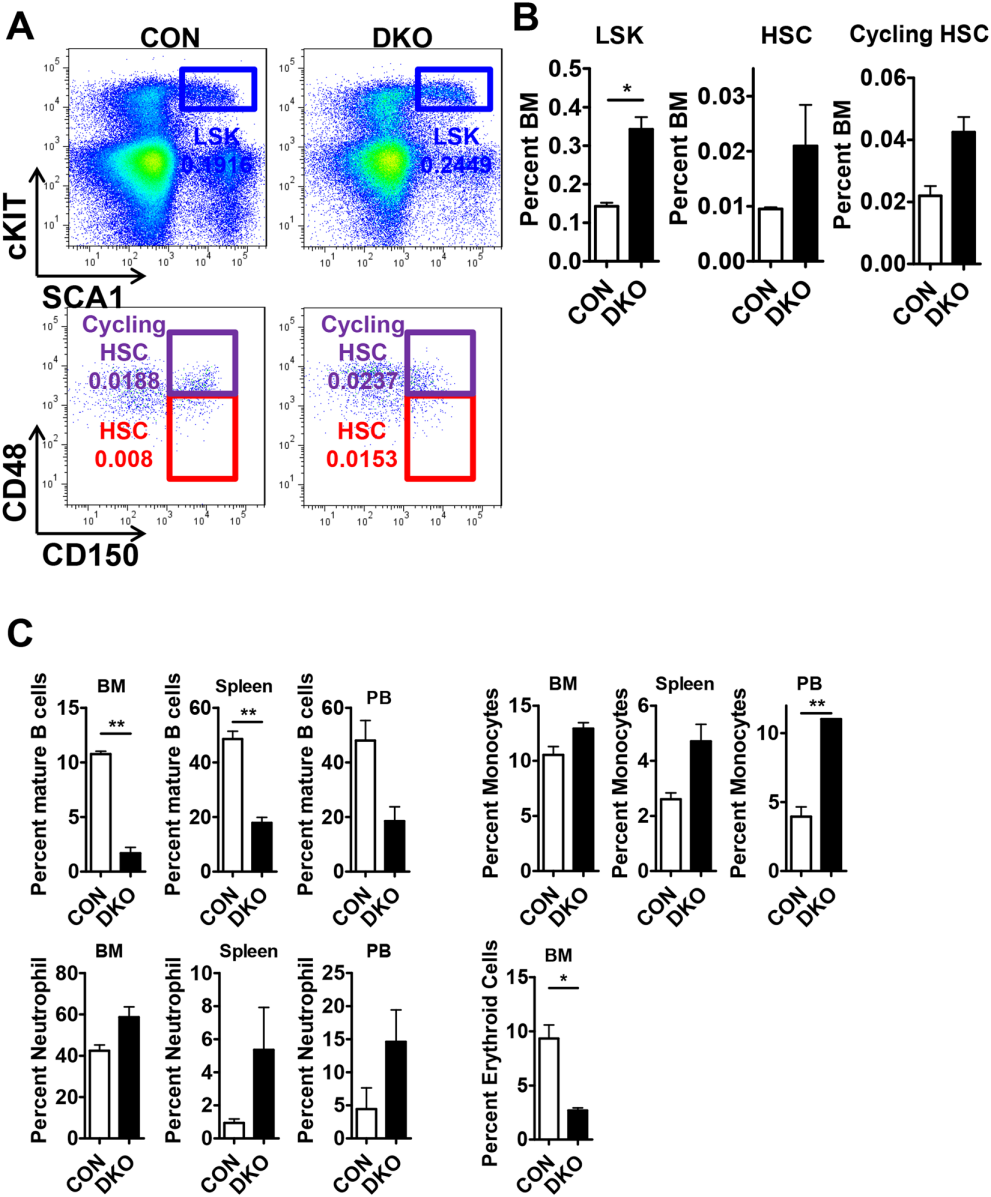
HSCs were not exhausted in 6-month-old *Mst1/2*-DKO mice. **(A)** FACS plot showing the frequency of LSK, HSC and cycling HSC populations. **(B)** The frequency of LSK cells, HSCs, and cycling HSCs was increased in *Mst1/2*-DKO cells. Error bars indicate S.E.M. (*p ≤ 0.05, n = 2). **(C)** The frequencies of B cells (B220) and myeloid cells (monocytes, Mac1^+^Gr1^lo^; neutrophils, Mac1^+^Gr1^+^) in the BM, spleen and PB, and erythroid cells (TER119) in the BM, from mice of the indicated *Mst1/2* genotypes are shown.

Increased proliferation of HSCs leads to stem cell exhaustion, as exemplified by conditional *Pten-*deficient mice, which show stem cell depletion within 6 weeks after induction of *Pten* knockout (pIpC injection)^25^. To determine whether stem cells are depleted in *Mst1/2*-deficient mice, we analyzed the frequency of cells in HSC compartments 6 months after induction of *Mst1/2* deletion by injection of pIpC (Fig. 3A and B). The frequencies of LSKs, HSCs and cycling HSCs were increased to levels similar to those in 3-week-old DKO mice, indicating that *Mst1/2* DKO does not cause stem cell exhaustion. These data suggest that expansion of HSC numbers continued after *Mst1/2* deletion and that the pools of these cells were maintained. Further, B cells were dramatically decreased in the BM, spleen and peripheral blood (PB) from in 6-month-old DKO mice as described before^26^ (Fig. 3C). We also found that DKO mice showed erythropenia in the BM and myeloid expansion in BM, spleen, and PB from in 6-month-old DKO mice. Collectively, we speculate that the reason why MST1/2 may expand HSC without exhaustion might be because MST1 and 2 modulate several functionally important targets in the other pathways that regulate an HSC program.

### MST1/2 are required for HSC function under conditions of stress

**Figure 4:**
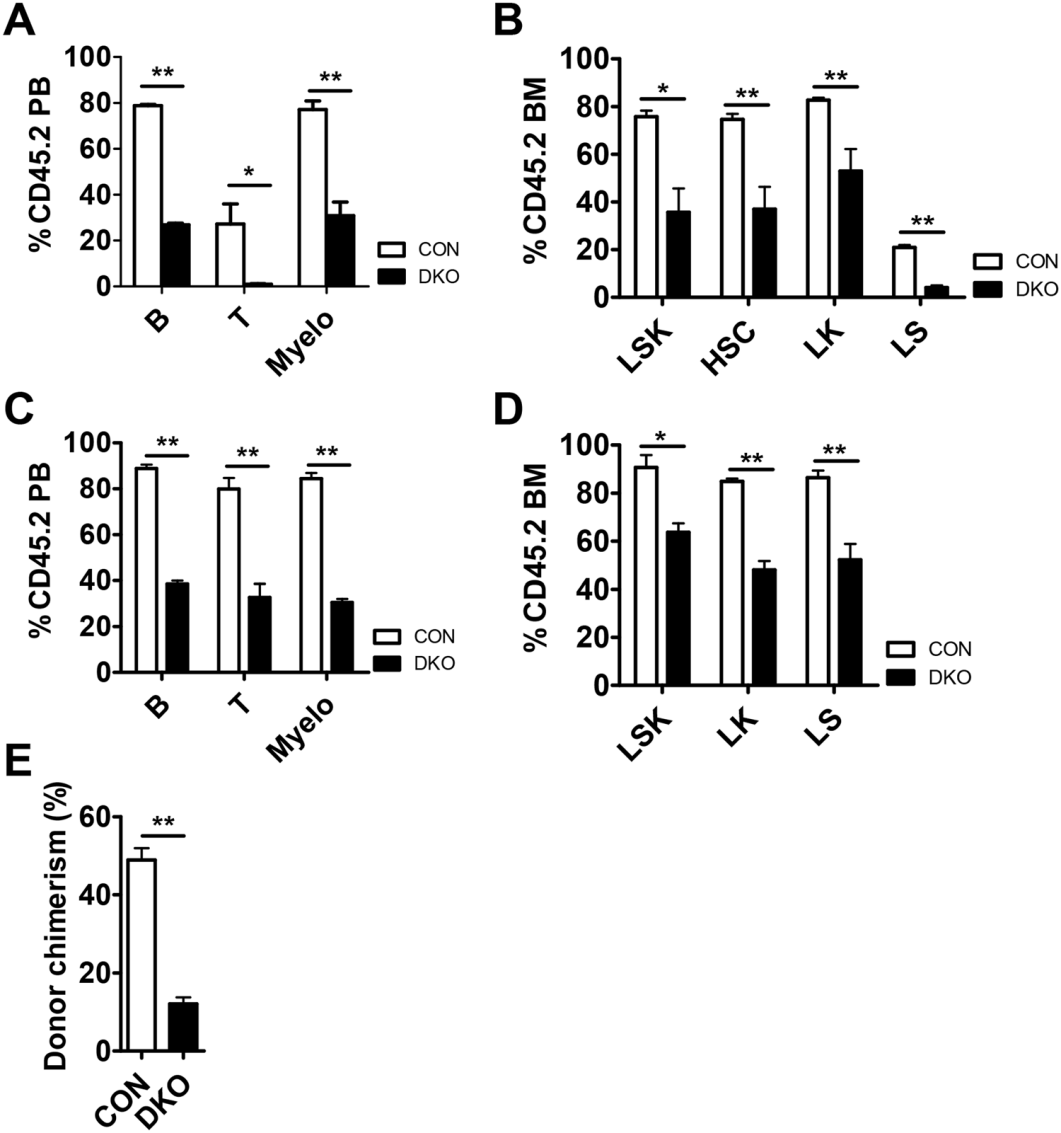
MST1/2 play important roles in HSC engraftment. **(A–D)** pIpC was injected to induce *Mx1-Cre* at 3 weeks before CD45.2 test cell isolation. A 4:1 mixture of the indicated CD45.2 test cells was transplanted together with CD45.1 competitor cells. PB was collected and analyzed for CD45.2^+^ chimerism at 4 weeks **(A** and **B)** and 4 months **(C** and **D)** post-transplantation. **(A** and **C)** The frequencies of B cells, T cells, and myeloid cells in PB from transplant recipients are shown. **(B** and **D)** The frequencies of LSK cells and HSCs in BM from transplant recipients are shown. **(E)** The graph shows frequencies of myeloid cells in PB from secondary transplant recipients. (*p ≤ 0.05, **p ≤ 0.01, n = 3–5).

To further test the function of *Mst1/2*-deficient HSCs, we transplanted BM cells from pIpC injected control or *Mst1/2*-DKO (CD45.2 cells) mice together with competitor cells into lethally irradiated CD45.1 mice. Competitors provided normal mature blood cells after reconstitution. Three weeks after transplantation, mice transplanted with *Mst1/2*-deficient cells displayed significantly diminished repopulation of mature blood cells compared with WT controls (Fig. 4A). This was caused by a decrease in HSCs and progenitor compartments of *Mst1/2*-deficient cells in transplanted recipient compared with control recipients (Fig. 4B). To examine donor chimerism of test cells following transplantation, we monitored repopulation maintenance in mice for 4 months. All cell lineages were detected throughout this period after transplantation, although the populations were lower than those in controls (Fig. 4C) and *Mst1/2*-deficient HSPCs were less well maintained at 4 months (Fig. 4D).

To determine whether *Mst1/2*-deficient HSCs are capable of surviving under secondary stress conditions, we performed a second transplantation of primary transplanted control and *Mst1/2*-deficient cells. We found that each hematopoietic cell lineage was less effectively reconstituted by *Mst1/2*-deficient HSCs. As was the case for primary transplantation, the reconstitution efficiency of secondarily transplanted *Mst1/2*-deficient cells was lower than that of control cells (Fig. 4E). These results indicate that MST1 and MST2 regulate BM engraftment efficiency.

### The reduced homing activity of *Mst1/2*-deficient cells results in defective engraftment

**Figure 5:**
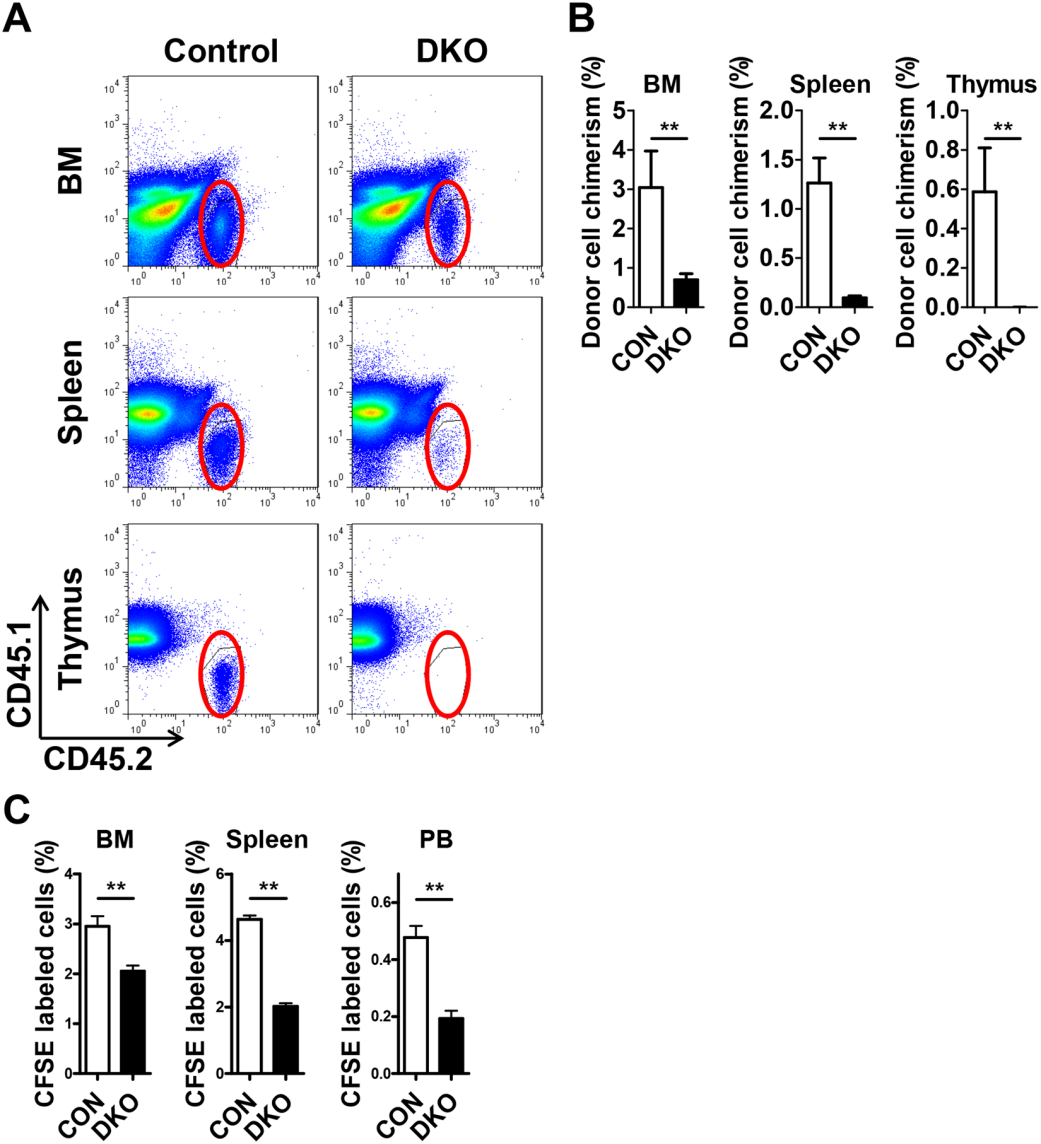
Short-term engraftment function of MST1/2 in acute transplantation stress. **(A** and **B)** The frequencies of *Mst1/2*-deleted donor cell chimerism in BM, spleen, and thymus of recipient mice at 3 weeks post-transplantation are shown. **(C)** The frequencies of *Mst1/2*-deleted donor cell populations in BM, spleen, and PB of recipient mice at 16 hours post-transplantation are shown. (**p ≤ 0.01, n = 3–7).

Engraftment efficiency can be determined by homing ability and/or proliferation/differentiation. Based on the increased HSC proliferation in *Mst1/2*-deficient mice, we suspected a defect in homing ability or engraftment efficiency of *Mst1/2*-deficient cells. To test short-term engraftment, we transplanted control or *Mst1/2*-deficient BM cells into non-irradiated CD45.1 recipient mice and examined donor cell chimerism in BM, spleen and thymus 3 weeks post-transplantation. *Mst1/2*-deleted HSPCs showed a decreased frequency of donor cell chimerism compared with controls (Fig. 5A,B). We further assessed the homing ability of *Mst1/2*-deficient BM cells by transplanting CFSE-labeled control or *Mst1/2*-deficient BM cells into lethally irradiated CD45.1 recipient mice and examining CFSE^+^ cells in BM, spleen and PB 16 hours post-transplantation. Fewer resident *Mst1/2*-deficient cells were found in BM, spleen, and PB of recipient mice compared with transplanted control cells (Fig. 5C). Collectively, these data suggest that the regulation of HSC engraftment by MST1 and MST2 is due, at least in part, to effects on the homing step.

## Discussion

In this study, we demonstrated that an *Mst1*/*2* deficiency leads to defects in the hematopoietic system. First, we found that *Mst1/2* deletion markedly altered mature cell output. Conditional *Mst1/2*-deficient mice exhibited severe B cell lymphopenia, and an increase in the myeloid cell population and mild erythropenia in BM, spleen, and PB. Second, we found that the loss of *Mst1/2* caused an increase in the steady state HSC population, a phenotype attributable to increased proliferation and reduced apoptosis of HSCs. However, stem cell exhaustion was not observed in 6-month-old *Mst1/2*-DKO mice, indicating that *Mst1/2*-deficient HSCs had expanded and were capable of maintaining their pools in the BM. Third, *Mst1/2* knockout in HSCs did not induce hyperplasia or spontaneous hematopoietic neoplasms up to the time of death (D.H. Lee, D. Lee and D.S. Lim, unpublished data).

We further found that the function of *Mst1/2*-deficient HSCs was impaired under stress conditions. *Mst1/2*-deficient recipient mice exhibited fewer mature cells in PB and significantly decreased HSC chimerism in BM compared with controls. Interestingly, *Mst1/2*-deleted BM cells showed a decreased frequency of donor cell chimerism and differences in their short-term engraftment (3 weeks) and homing (16 hours) ability compared with controls under acute transplantation stress conditions. These data suggest that the inefficient engraftment of *Mst1/2*-deficient BM cells in the acute stress transplantation setting is attributable to decreased homing ability. Collectively, our findings suggest that Mst1 and Mst2 regulate HSC function in a setting of acute stress.

Numerous studies have reported that MST1 and MST2 are important in T cell migration^18,27,28^. By extension, it is reasonable to suggest that MST1 and MST2 are also essential for HSC migration into BM and adhesion to the BM microenvironment. Consistent with this, *Mst1/2*-deficient HSCs accumulated abnormally in PB and spleen, a phenomenon caused by defective HSC retention in the BM niche. Accordingly, we suggest that MST1 and MST2 will prove to be key factors for improving future human HSC transplantation therapies using cord blood or adult blood. In summary, MST1 and MST2 control the production of mature hematopoietic cells and play an indispensable role in the maintenance and function of hematopoietic stem and progenitor cells.

## Electronic supplementary material

Supplementary information
