# Supplementary material for: Mammalian sterile 20 kinase 1 and 2 are important regulators of hematopoietic stem cells in stress condition
Source: Sci Rep. 2018 Jan 17;8:942. doi: 10.1038/s41598-018-19637-y (PMC5772645; doi:10.1038/s41598-018-19637-y)

Mammalian sterile 20 kinase 1 and 2 are important regulators of hematopoietic stem cells in stress condition

Da-Hye Lee[1], Tae-Shin Kim[1], Dongjun Lee[2,*], Dae-Sik Lim[1,*]

[1]Department of Biological Sciences, National Creative Research Initiatives Center, Korea Advanced Institute of Science and Technology, Daejeon, Korea

[2]Department of Medical Science, Pusan National University School of Medicine, Yangsan, Korea

*Corresponding author: Dongjun Lee (lee.dongjun@pusan.ac.kr) and Dae-Sik Lim (daesiklim@kaist.ac.kr)

The authors declare that no conflicts of interest exist.

Supplemental Figure 1

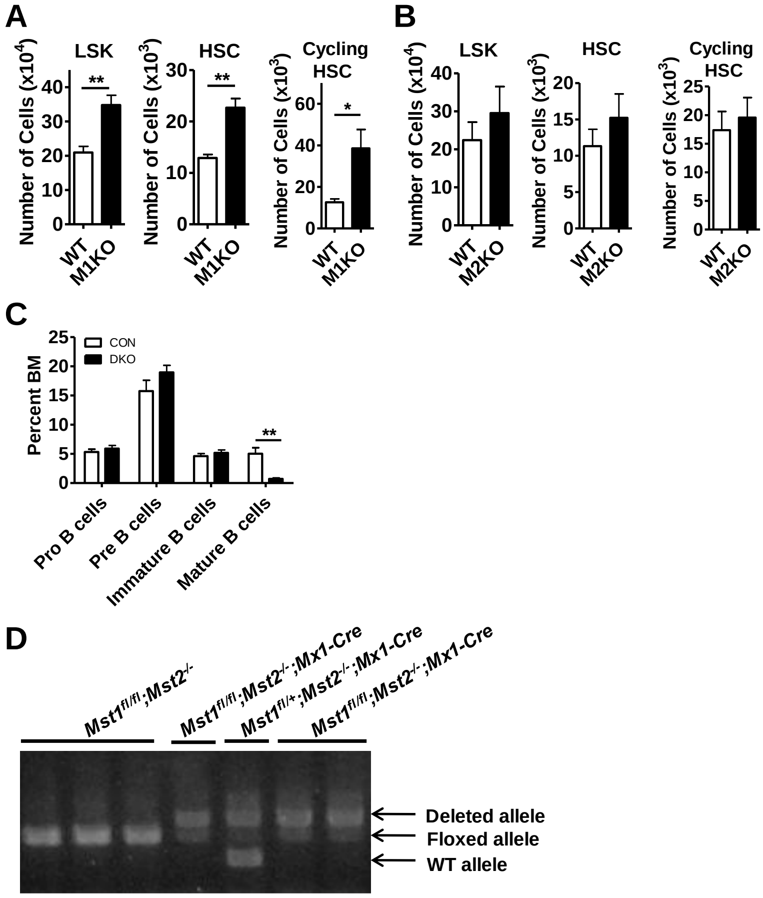

Figure S1. The numbers of HSCs in BM of **(A)** *Mst1*-deficient and **(b)** *Mst2*-deficient mice are shown (*p ≤ 0.05, **p ≤ 0.01, n = 3–5). **(C)** The frequencies of differential B cell population in the BM are shown from mice of the indicated *Mst1/2* genotypes are shown (*p ≤ 0.05, **p ≤ 0.01, n = 4). **(D)** PCR was performed on DNA isolated from PB to assess allele recombination from cells of the indicated genotype.

Supplemental Figure 2

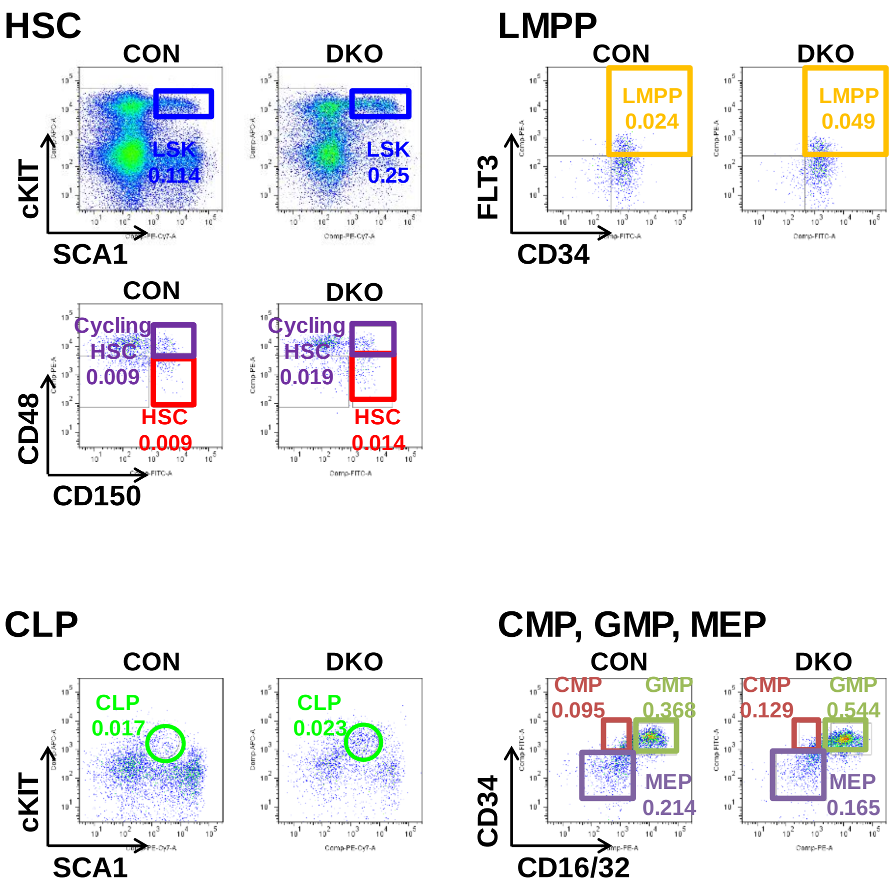

Figure S2. Representative FACS plots showing the frequency of LSK, HSC and cycling HSC populations.

Uncropped gel image

Supplementary figure 1D

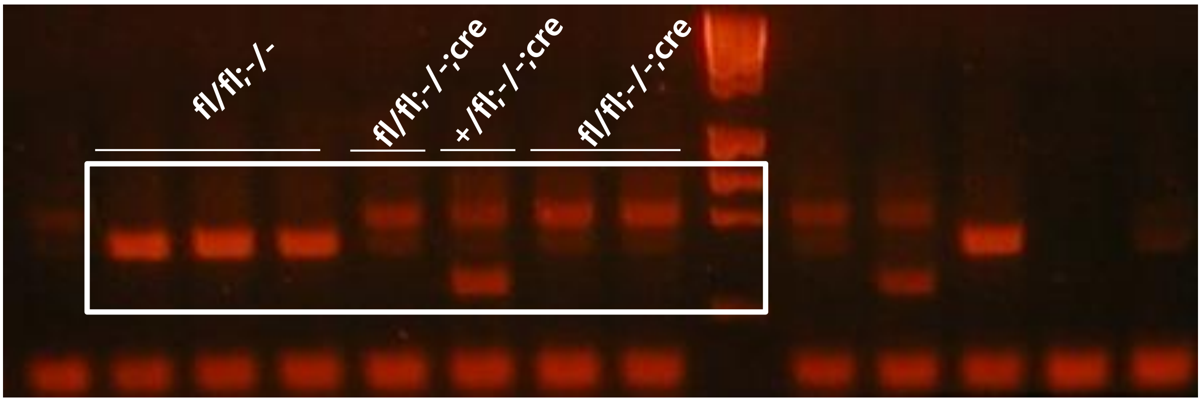

Supplement: Supplementary file 1 — Supplementary information [file 41598_2018_19637_MOESM1_ESM.pdf]
